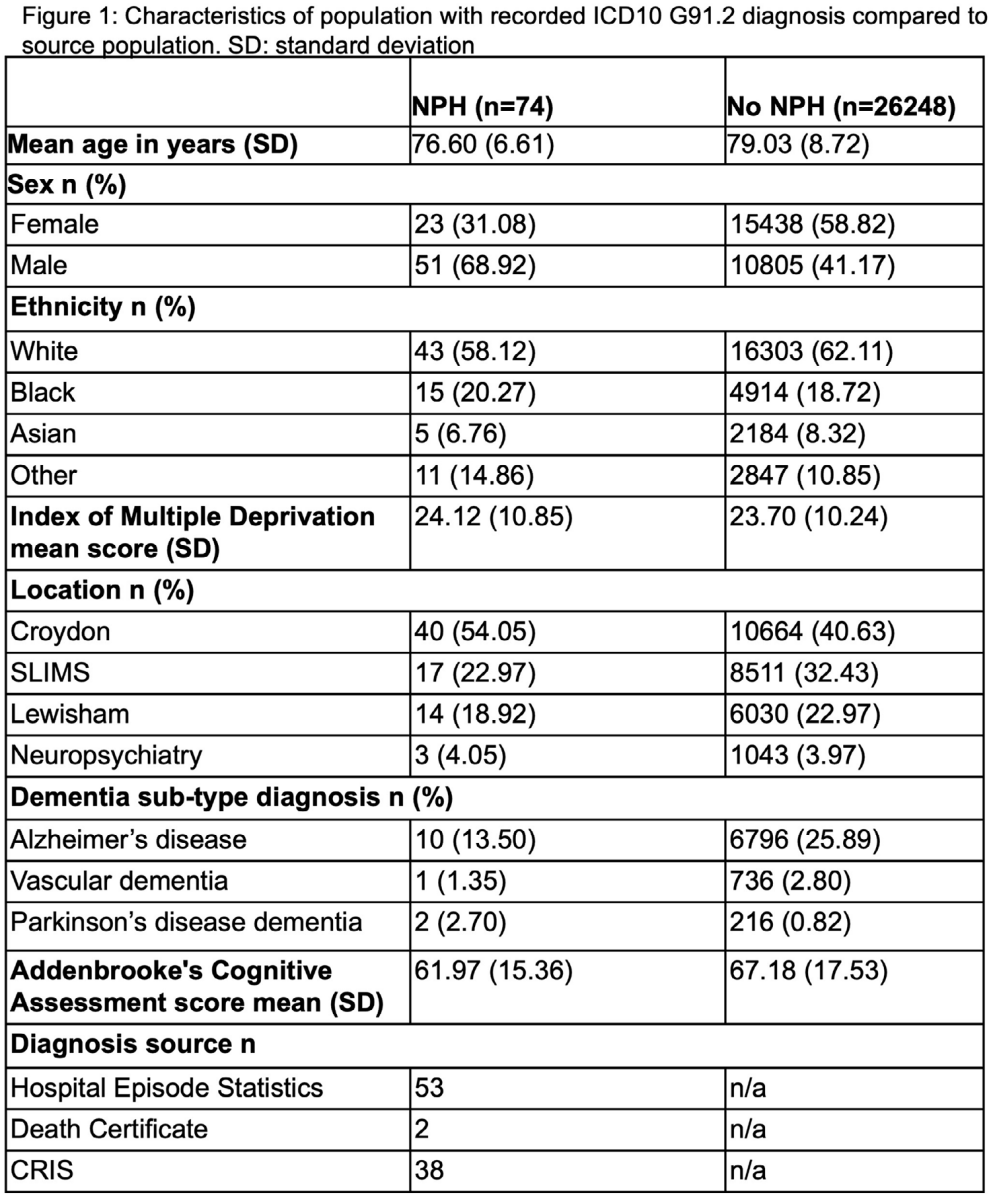# Characteristics of diagnosed normal pressure hydrocephalus in routinely‐collected data from memory assessment services in a large South London catchment area

**DOI:** 10.1002/alz70861_108354

**Published:** 2025-12-23

**Authors:** Clara Belessiotis‐Richards, Gill Livingston, Hitesh Shetty, Robert Stewart

**Affiliations:** ^1^ Department of Psychological Medicine, Institute of Psychiatry, Psychology and Neuroscience, Kings' College London, London UK; ^2^ Psychiatry, University College London, London, London UK; ^3^ North London NHS Foundation trust, London, London UK; ^4^ South London and the Maudsley NHS Foundation Trust, London UK; ^5^ Department of Psychological Medicine, King's College London, London UK; ^6^ NIHR Biomedical Research Centre for Mental Health and Biomedical Research Unit for Dementia at South London and Maudsley NHS Foundation, London UK

## Abstract

**Background:**

Normal pressure hydrocephalus (NPH) is a treatable but potentially under‐diagnosed condition causing dementia. At presentation, half of people with NPH score in the dementia range on formal testing, and 75% have cognitive impairment. However, little is known about NPH in memory services. Here, we describe recorded NPH in a large, ethnically and socioeconomically diverse memory service cohort for the first time.

**Method:**

South London and Maudsley (SLAM) NHS Trust in England has a catchment of 1.3 million residents. All people aged 60 years and above referred to memory services in SLAM from 01/01/2008 to 22/02/2025 were included. We extracted data on age, sex, ethnicity, neighbourhood‐level deprivation, cognitive test results, dementia diagnosis, and location from SLAM’s Clinical Record Interactive Search (CRIS) system, national death certification data, and from Hospital Episode Statistics (HES), which records data on all hospital diagnoses in England. Diagnosis of NPH was defined as any recorded code of ICD10 G91.2.

**Result:**

74 diagnoses of G91.2 were recorded (*n* =26,322). Mean age was lower in people with recorded NPH (76.60 years, standard deviation (SD): 6.61), compared to the source cohort (79.03, SD: 8.72) (see Figure 1). More people with recorded NPH were male (68.92%) compared to the source memory clinic population (41.17%). Mean Addenbrookes’ Cognitive Examination (ACE) score was lower among people with recorded NPH (61.97, SD: 15.36) than the source cohort (67.18, SD: 17.53).

**Conclusion:**

Our findings could reflect under‐diagnosis of NPH in memory services. Recorded diagnosis of NPH in our population was rare, with a 17‐year case proportion of 0.28%. Other studies have estimated the point prevalence of NPH in the general population above 60 years old to be 0.45% and, though our measure is not directly comparable, we expected a higher number of cases in this clinical population with cognitive symptoms. We focussed on recorded diagnosis of G91.2, which may have limited our results due to inconsistent diagnostic code use. These findings warrant further work including investigating the chronology of diagnosis with memory clinic referral and investigation of alternative ICD10 codes used for NPH in this population.